# Quantification and accurate normalisation of small RNAs through new custom RT-qPCR arrays demonstrates *Salmonella*-induced microRNAs in human monocytes

**DOI:** 10.1186/1471-2164-13-23

**Published:** 2012-01-16

**Authors:** Soroush Sharbati, Jutta Sharbati, Lena Hoeke, Marc Bohmer, Ralf Einspanier

**Affiliations:** 1 Institute of Veterinary Biochemistry, Freie Universitaet Berlin, Berlin, Germany

**Keywords:** microRNA, RT-qPCR, array, normalisation, isomiR, *Salmonella*, macrophage

## Abstract

**Background:**

Small interfering and non-coding RNAs regulate gene expression across all kingdoms of life. MicroRNAs constitute an important group of metazoan small RNAs regulating development but also disease. Accordingly, in functional genomics microRNA expression analysis sheds more and more light on the dynamic regulation of gene expression in various cellular processes.

**Results:**

We have developed custom RT-qPCR arrays allowing for accurate quantification of 31 small RNAs in triplicate using a 96 well format. In parallel, we provide accurate normalisation of microRNA expression data based on the quantification of 5 reference snRNAs. We have successfully employed such arrays to study microRNA regulation during human monocyte differentiation as well as *Salmonella *infection. Besides well-known protagonists such as miR-146 or miR-155, we identified the up-regulation of miR-21, miR-222, miR-23b, miR-24, miR-27a as well as miR-29 upon monocyte differentiation or infection, respectively.

**Conclusions:**

The provided protocol for RT-qPCR arrays enables straight-forward microRNA expression analysis. It is fully automatable, compliant with the MIQE guidelines and can be completed in only 1 day. The application of these arrays revealed microRNAs that may mediate monocyte host defence mechanisms by regulating the TGF-β signalling upon *Salmonella *infection. The introduced arrays are furthermore suited for customised quantification of any class of small non-coding RNAs as exemplified by snRNAs and thus provide a versatile tool for ubiquitous applications.

## Background

Metazoan regulation of gene expression relies on an interwoven network of e.g. DNA methylation, transcription factors, mRNA degradation or translational control. Recent research has shown that translational regulation as well as mRNA degradation is controlled by RNA interference (RNAi). The main class of intrinsic small regulating RNAs concerting these effects in eukaryotes is constituted of microRNAs (miRNAs). Mature miRNAs (about 20 nt length) derive from a hairpin. The active strand is loaded to an Argonaute family protein to form the miRNA induced silencing complex (miRISC), which recognises the target site within a 3' UTR. Animal miRISC was originally thought to repress target translation rather than mRNA degradation. However, recent data suggest that mRNA degradation may be the predominant mode of miRNA mediated regulation of gene expression [1]. Accordingly, various studies have shown negatively correlated expression of miRNAs and their targets [2,3]. Based on these observations, in silico tools such as MAGIA [4] were developed to link target prediction to the expression analysis of miRNAs and their target mRNAs. Connecting negatively correlated miRNA and target mRNA expression with target prediction allows for the identification of aberrations in miRNA mediated regulation among various disease related pathways. The role of miRNA mediated gene regulation in development and disease such as cancer or viral infections was recognised very early. However, very recent studies suggest that miRNAs are also involved in the specific host response to bacterial pathogens such as *Mycobacteria *or *Salmonella *[5-8]. In this regard, integrated miRNA- as well as mRNA-transcriptome analysis by means of microarrays and reverse transcription quantitative PCR (RT-qPCR) allowed us to show that in mycobacterial infections of human macrophages caspases 3 and 7 are under the control of let-7e and miR-29a, respectively [8].

Many methods such as microarrays, RNAseq or RT-qPCR are used to study mRNA as well as miRNA expression of cells or tissues under a given condition. Quantification of miRNAs but also other small non-coding RNAs by means of RT-qPCR was established during the last decade based on several detection strategies. In this regard, we developed a protocol called miR-Q, which relies on reverse transcription (RT) of miRNAs using specific oligonucleotides. The generated cDNA is quantified by means of a novel qPCR protocol using three oligonucleotides based on SYBR Green detection chemistry [9]. The protocol can easily be customised to detect and quantify any class of non-coding small RNAs. Besides the frequent application of miR-Q in our lab, the protocol was adopted by others for quantification of small RNAs e.g. in immunity-, virus-, metabolism- or cancer-related RNAi research [10-13]. The miR-Q protocol enables highly specific quantification of single miRNAs providing a high sensitivity. Since RT-qPCR remains the gold-standard for accurate quantification of gene expression, such arrays are increasingly employed for mid-throughput quantification of gene expression providing high sensitivity coupled with accuracy. While arrayed quantification of mRNAs is performed easily, the short length of miRNAs and underlying detection chemistries require well-conceived strategies. A few commercial miRNA RT-qPCR arrays are currently available, which are rather costly. Based on the stem-looped qPCR approach of Applied Biosystems Tang and colleagues reported the 220-plex miRNA expression of a single cell [14]. Another group reported a multiplex quantification of miRNAs involving the purification of multiplex PCR amplicons followed by uniplex analysis on microfluidic chips [15]. However, until today there are no non-commercial and custom protocols for miRNA RT-qPCR arrays. This prompted us to advance the existing miR-Q protocol allowing more versatile and fully automatable arrayed quantification of any class of eukaryotic small non-coding RNAs. Compliant with the MIQE guidelines and nomenclature [16], we provide here a reliable and cost-effective method enabling arrayed quantification combined with accurate normalisation of miRNA expression.

## Results and discussion

### Multiplexed reverse transcription of miRNAs enables miR-Q arrays

**Figure 1:**
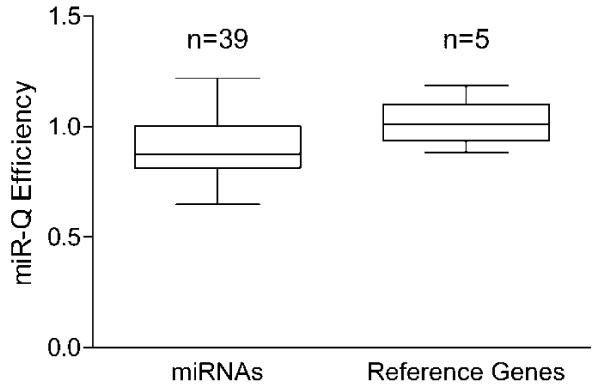
**Efficiency of miR-Q assays**. The Tukey box plots show the efficiencies of 39 miRNA- as well as 5 reference snRNA-specific miR-Q assays. A paired t test proved that there was no statistically significant difference between the efficiencies of miRNA- and reference snRNA-specific miR-Q assays (P = 0.6836).

After the miR-Q approach was established and published, several labs worldwide approved its performance for their research by adopting the protocol. Over the course of the last three years we further established miR-Q assays for several miRNAs according to different research projects [8,17,18]. Another 21 newly established assays are presented here for the first time including a set of five snRNA specific assays serving as reference genes. Taken together, we have developed 44 small RNA specific miR-Q assays and the particular oligonucleotide sequences are presented in table 1. As reported earlier [9], we usually determine the dynamic range of miR-Qs by spiking bacterial total RNA with serial dilutions of synthetic miRNA in single assays (2 nM-2 fM final concentration regarding the RT-qPCR). Most miR-Q assays possess a dynamic range of 5 to 6 orders of magnitude. According to our experience the lower spectrum of calibration curves however corresponds to the naturally occurring and physiological miRNA concentration of a cell. Therefore, we determined the Y-intercept, slope and r^2 ^for all presented miRNA specific assays ranging from 20 pM to 20 nM (Additional file 1). The dynamic range of reference snRNA specific assays was determined in a range between 100 and 0.01 ng cDNA. As it is shown in Figure 1, all assays possessed proper efficiencies calculated from the slopes of calibration curves. Figure 1 moreover shows that there was no statistically significant difference between the efficiencies of miRNA- and reference snRNA-specific miR-Q assays proven by a paired t test (P = 0.6836).

**Table 1:** Oligonucleotides for miR-Q arrays.

miRNA	RT6-'miRNA'	'miRNA'-rev	**Ref**.
*let-7a*	*tgtcaggcaaccgtattcacc*gtgagtggt**aactat**	*cgtcagatgtccgagtagagg*gggaacggcg**tgaggtagtaggttgtata**	[9]
*let-7b*	*tgtcaggcaaccgtattcacc*gtgagtggt**aaccac**	*cgtcagatgtccgagtagagg*gggaacggcg**tgaggtagtaggttgtgtg**	[9]
*let-7c*	*tgtcaggcaaccgtattcacc*gtgagtggt**aaccat**	*cgtcagatgtccgagtagagg*gggaacggcg**tgaggtagtaggttgtatg**	[9]
*let-7e*	*tgtcaggcaaccgtattcacc*gtgagtggt**aactat**	*cgtcagatgtccgagtagagg*gggaacggcg**tgaggtaggaggttgt**	[8]
*let-7f*	*tgtcaggcaaccgtattcacc*gtgagtggt**actata**	*cgtcagatgtccgagtagagg*gggaacggcg**tgaggtagtagattgtat**	This study
*miR-103a*	*tgtcaggcaaccgtattcacc*gtgagtggt**tcatag**	*cgtcagatgtccgagtagagg*gggaacggcg**agcagcattgtacaggg**	[17]
*miR-106a*	*tgtcaggcaaccgtattcacc*gtgagtggt**tacctg**	*cgtcagatgtccgagtagagg*gggaacggcg**aaaagtgcttacagtg**	This study
*miR-141*	*tgtcaggcaaccgtattcacc*gtgagtggt**ccatct**	*cgtcagatgtccgagtagagg*gggaacggcg**taacactgtctggtaaag**	[9]
*miR-143*	*tgtcaggcaaccgtattcacc*gtgagtggt**tgagct**	*cgtcagatgtccgagtagagg*gggaacggcg**tgagatgaagcactgt**	This study
*miR-145*	*tgtcaggcaaccgtattcacc*gtgagtggt**aaggga**	*cgtcagatgtccgagtagagg*gggaacggcg**gtccagttttcccaggaa**	[9]
*miR-146a*	*tgtcaggcaaccgtattcacc*gtgagtggt**aaccca**	*cgtcagatgtccgagtagagg*gggaacggcg**tgagaactgaattcca**	[8]
*miR-146b-5p*	*tgtcaggcaaccgtattcacc*gtgagtggt**agccta**	*cgtcagatgtccgagtagagg*gggaacggcg**tgagaactgaattccata**	[8]
*miR-148a*	*tgtcaggcaaccgtattcacc*gtgagtggt**acaaag**	*cgtcagatgtccgagtagagg*gggaacggcg**tcagtgcactacagaa**	This study
*miR-155*	*tgtcaggcaaccgtattcacc*gtgagtggt**acccct**	*cgtcagatgtccgagtagagg*gggaacggcg**ttaatgctaatcgtgat**	[8]
*miR-15b*	*tgtcaggcaaccgtattcacc*gtgagtggt**tgtaaa**	*cgtcagatgtccgagtagagg*gggaacggcg**tagcagcacatcatggttt**	This study
*miR-16*	*tgtcaggcaaccgtattcacc*gtgagtggt**cgccaa**	*cgtcagatgtccgagtagagg*gggaacggcg**tagcagcacgtaaata**	[9]
*miR-181a*	*tgtcaggcaaccgtattcacc*gtgagtggt**caccga**	*cgtcagatgtccgagtagagg*gggaacggcg**aacattcaacgctg**	[9]
*miR-191*	*tgtcaggcaaccgtattcacc*gtgagtggt**agctgc**	*cgtcagatgtccgagtagagg*gggaacggcg**caacggaatcccaaaa**	This study
*miR-200c*	*tgtcaggcaaccgtattcacc*gtgagtggt**ccatca**	*cgtcagatgtccgagtagagg*gggaacggcg**taatactgccgggtaa**	[9]
*miR-21*	*tgtcaggcaaccgtattcacc*gtgagtggt**tcaaca**	*cgtcagatgtccgagtagagg*gggaacggcg**tagcttatcagactga**	[9]
*miR-214*	*tgtcaggcaaccgtattcacc*gtgagtggt**actgcc**	*cgtcagatgtccgagtagagg*gggaacggcg**acagcaggcacagacagg**	This study
*miR-222*	*tgtcaggcaaccgtattcacc*gtgagtggt**gagacc**	*cgtcagatgtccgagtagagg*gggaacggcg**agctacatctggctactg**	This study
*miR-22-3p*	*tgtcaggcaaccgtattcacc*gtgagtggt**acagtt**	*cgtcagatgtccgagtagagg*gggaacggcg**aagctgccagttgaagaac**	This study
*miR-23b*	*tgtcaggcaaccgtattcacc*gtgagtgct**ggtaat**	*cgtcagatgtccgagtagagg*gggaacggcg**atcacattgccaggg**	[9]
*miR-24*	*tgtcaggcaaccgtattcacc*gtgagtggt**ctgttc**	*cgtcagatgtccgagtagagg*gggaacggcg**tggctcagttcagcag**	[9]
*miR-27a*	*tgtcaggcaaccgtattcacc*gtgagtggt**gcggaa**	*cgtcagatgtccgagtagagg*gggaacggcg**ttcacagtggctaag**	[9]
*miR-296-3p*	*tgtcaggcaaccgtattcacc*gtgagtggt**aggarag**	*cgtcagatgtccgagtagagg*gggaacggcg**gagggttgggtggaggc**	This study
*miR-29a*	*tgtcaggcaaccgtattcacc*gtgagtggt**taaccg**	*cgtcagatgtccgagtagagg*gggaacggcg**tagcaccatctgaaatcgg**	[8]
*miR-29b*	*tgtcaggcaaccgtattcacc*gtgagtggt**aacact**	*cgtcagatgtccgagtagagg*gggaacggcg**tagcaccatttgaaatcagt**	This study
*miR-29c*	*tgtcaggcaaccgtattcacc*gtgagtggt**taaccg**	*cgtcagatgtccgagtagagg*gggaacggcg**tagcaccatttgaaatcgg**	This study
*miR-30a*	*tgtcaggcaaccgtattcacc*gtgagtggt**cttcca**	*cgtcagatgtccgagtagagg*gggaacggcg**tgtaaacatcctcgactgg**	This study
*miR-30b*	*tgtcaggcaaccgtattcacc*gtgagtggt**agctga**	*cgtcagatgtccgagtagagg*gggaacggcg**tgtaaacatcctacactca**	This study
*miR-30c*	*tgtcaggcaaccgtattcacc*gtgagtggt**gctgag**	*cgtcagatgtccgagtagagg*gggaacggcg**tgtaaacatcctacactctc**	This study
*miR-326*	*tgtcaggcaaccgtattcacc*gtgagtggt**ctggag**	*cgtcagatgtccgagtagagg*gggaacggcg**cctctgggcccttc**	[9]
*miR-371-5p*	*tgtcaggcaaccgtattcacc*gtgagtggt**agtgcc**	*cgtcagatgtccgagtagagg*gggaacggcg**actcaaactgtggg**	This study
*miR-423-3p*	*tgtcaggcaaccgtattcacc*gtgagtggt**tgaggg**	*cgtcagatgtccgagtagagg*gggaacggcg**agctcggtctgaggc**	[9]
*miR-451*	*tgtcaggcaaccgtattcacc*gtgagtggt**aaactc**	*cgtcagatgtccgagtagagg*gggaacggcg**aaaccgttaccattactgag**	[9]
*miR-484*	*tgtcaggcaaccgtattcacc*gtgagtggt**atcggg**	*cgtcagatgtccgagtagagg*gggaacggcg**tcaggctcagtcccct**	[9]
*miR-886-5p*	*tgtcaggcaaccgtattcacc*gtgagtggt**ccgctt**	*cgtcagatgtccgagtagagg*gggaacggcg**cgggtcggagttagctc**	[8]
*RNU6*	*tgtcaggcaaccgtattcacc***aaaaatat**	*cgtcagatgtccgagtagagg***gtgctcgcttcggcagc**	This study
*SNORD44*	*tgtcaggcaaccgtattcacc***agtcag**	*cgtcagatgtccgagtagagg***cctggatgatgataagcaaatg**	This study
*SNORD47*	*tgtcaggcaaccgtattcacc***aacctc**	*cgtcagatgtccgagtagagg***aaccaatgatgtaatgattctgc**	This study
*SNORD48*	*tgtcaggcaaccgtattcacc***ggtcag**	*cgtcagatgtccgagtagagg***agtgatgatgaccccaggta**	This study
*SNORD52*	*tgtcaggcaaccgtattcacc***tcagaa**	*cgtcagatgtccgagtagagg***gggaatgatgatttcacagact**	This study

The table comprises oligonucleotides for reverse transcription (RT6-'miRNA') as well as the specific primers for qPCR detection ('miRNA'-rev). MiRNA-specific nucleotides are indicated in bold letters, while binding sites of terminal primers (MP-fw: tgtcaggcaaccgtattcacc and MP-rev: cgtcagatgtccgagtagagg) are indicated in italic.

**Figure 2:**
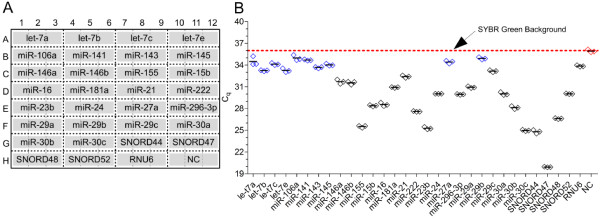
**Arrayed quantification of miRNAs using a 96 well format**. A miR-Q array layout is exemplified in Figure 2A using a 96-well format. This layout provides triplicate quantification of 26 miRNAs, 5 reference snRNAs and negative controls (NC). Figure 2B shows the C_q _variations of triplicate measurements using 1 μg total RNA from differentiated THP-1. Rhombi indicate the individual measurements of each assay, while their mean is given as black lines. Black rhombi represent assays possessing specific products and blue rhombi show unspecific detection. The SYBR Green background is given as red dotted line.

According to our experience, methods such as microarrays or next-generation-sequencing can be applied for the generation of snap shots of a cell or tissue miRNAome [8,19]. However, we were interested in a fast, cost-effective, feasible and accurate alternative. Accordingly, we advanced the conventional miR-Q protocol and reaction conditions allowing increased performance of miRNA RT-qPCR arrays (miR-Q arrays). For this purpose, we evaluated multiplexed RT reactions with different numbers of specific RT oligonucleotides per reaction (RT6-'miRNA') and varying respective final concentrations. In contrast to the conventional miR-Q RT, the reaction time was extended and a higher amount of reverse transcriptase was used for the miR-Q arrays. A 31-plex RT turned out to be most suitable because it allowed detection of the respective number of small RNAs and negative controls (NC) measured in triplicate when using a 96-well format. A miR-Q array layout is exemplified in Figure 2A. This layout allowed the detection of 26 different miRNAs within the same PCR run and simultaneously quantifying the expression of 5 additional reference snRNAs (SNORD44, 47, 48, 52 and RNU6) serving for normalisation of miRNA expression. Differently composed layouts were performed resulting in no composition-based bias (data not shown). Multiplexed RT reactions furthermore provide harmonised conversion efficiency of miRNAs as well as reference snRNAs and are a prerequisite for reliable normalisation of expression.

We used total RNA isolated from differentiated human THP-1 to prove the repeatability of miR-Q arrays. Figure 2B shows the triplicate C_q _values using the layout shown in Figure 2A. Except for 10 miRNAs (let-7 family, miR-106a, miR-141, miR-143, miR-145, miR-27a and miR-29b) all other miRNAs and reference snRNAs were detected in differentiated THP-1 showing minor C_q _variation in triplicate measurements and demonstrating strong repeatability of miR-Q arrays (Figure 2B). Commercially available RT-qPCR arrays often employ single measurement for individual miRNAs, so that the lack of intra-array replicates weakens the repeatability of assessed data. Furthermore, there is no opportunity to evaluate if an undetermined C_q _is based on target absence or failed reaction. For generation of highly reliable results we encourage intra-array triplicate measurements of each target.

### A set of 5 snRNAs allows for accurate normalisation of miRNA expression

**Figure 3:**
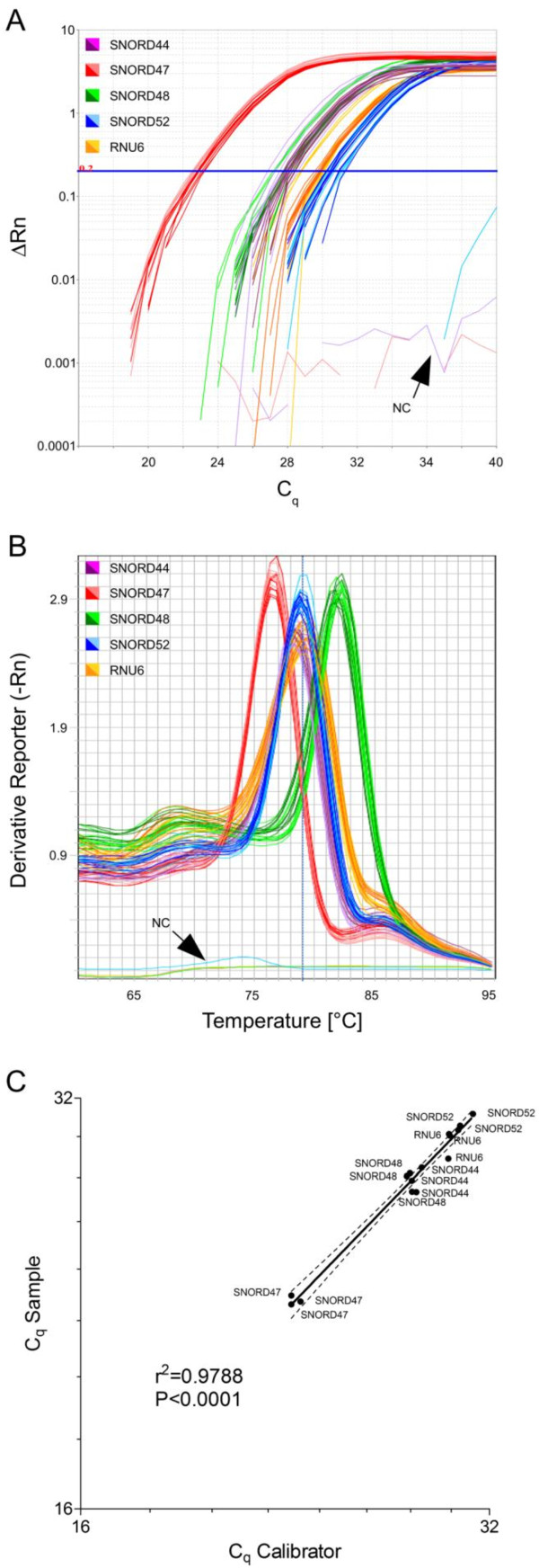
**Normalisation of miR-Q arrays using a set of 5 reference snRNAs**. Triplicate detection of reference snRNAs (SNORD44, SNORD47, SNORD48, SNORD52 and RNU6) in human primary macrophages of three independent donors that were infected (samples) with *Mycobacterium avium hominissuis *or remained non-infected (calibrators) is shown Figure 3A. The measurement revealed minor C_q _variation in samples and calibrators while all negative controls (NC) remained negative across 40 cycles. Light colours represent the infected samples and dark colours the non-infected calibrators. Figure 3B shows melting curves proving the specificity of all amplicons. Dissociation analysis was performed by initial denaturation at 95°C for 1 min, amplicon hybridisation at 60°C for 2 min and melting by ramping from 60°C to 95°C at 2°C/min and acquiring the fluorescence signal. Figure 3C evaluates the stability of the chosen reference snRNAs among donors as well as samples (infected) and calibrators (non-infected) as demonstrated by the coefficient of determination r^2 ^= 0.9788 (P < 0.0001).

Accurate normalisation of gene expression measures is a crucial process that controls for bias based on extraction yield, RT yield and amplification efficiency. This step is the basic prerequisite for comparing gene expression values among different samples [16]. For normalisation of miR-Q array data, we have designed assays for a set of 5 snRNAs: SNORD44, SNORD47, SNORD48, SNORD52 and RNU6. We tested for applicability of these reference snRNAs using human primary macrophages from three independent donors that were infected with *Mycobacterium avium hominissuis *or remained non-infected described in a previous study [8]. As shown in Figure 3A, all reference snRNAs were detected among the three donors showing minor C_q _variation in samples (infected) and calibrators (non-infected) while all negative controls (NC) remained negative across 40 cycles. Melting curve analysis proved all amplicons to be specific (Figure 3B) and RT negative controls revealed the absence of contaminating genomic DNA (data not shown). For evaluating the stability of the chosen reference snRNAs among donors as well as samples and calibrators, we have plotted C_q _values of samples versus calibrators (Figure 3C). The correlation analysis revealed high linearity between C_q sample _and C_q calibrator _possessing a coefficient of determination close to 1 (r^2 ^= 0.9788 and P < 0.0001) and pointing to excellent stability of all reference snRNA. This result was also verified by calculating the geNorm stability measure M [20]. For this purpose, fold differences were calculated from ΔC_q _between samples and calibrators. The calculated M values for the entire set were below 0.5 (M_SNORD44 _= 0.37; M_SNORD47 _= 0.41; M_SNORD48 _= 0.44; M_SNORD52 _= 0.3; M_RNU6 _= 0.31) pointing also to high stability of presented reference genes and underlining the correlation analysis. This set of 5 reference snRNAs was also highly stable in other macrophages as well as intestinal samples (data not shown). For comparative quantification analysis based on the ΔΔC_q _algorithm we emphasise the evaluation of the stability of reference snRNAs in the studied samples as described above. The geometric mean of the most stable candidates among the set of 5 can then be chosen for normalisation of miRNA expression values (ΔC_q _= C_q miRoI _- C_q norm_). We encourage the use of at least 3 reference snRNAs for normalisation of miRNA expression.

### miR-Q arrays are highly reproducible and accurate

**Figure 4:**
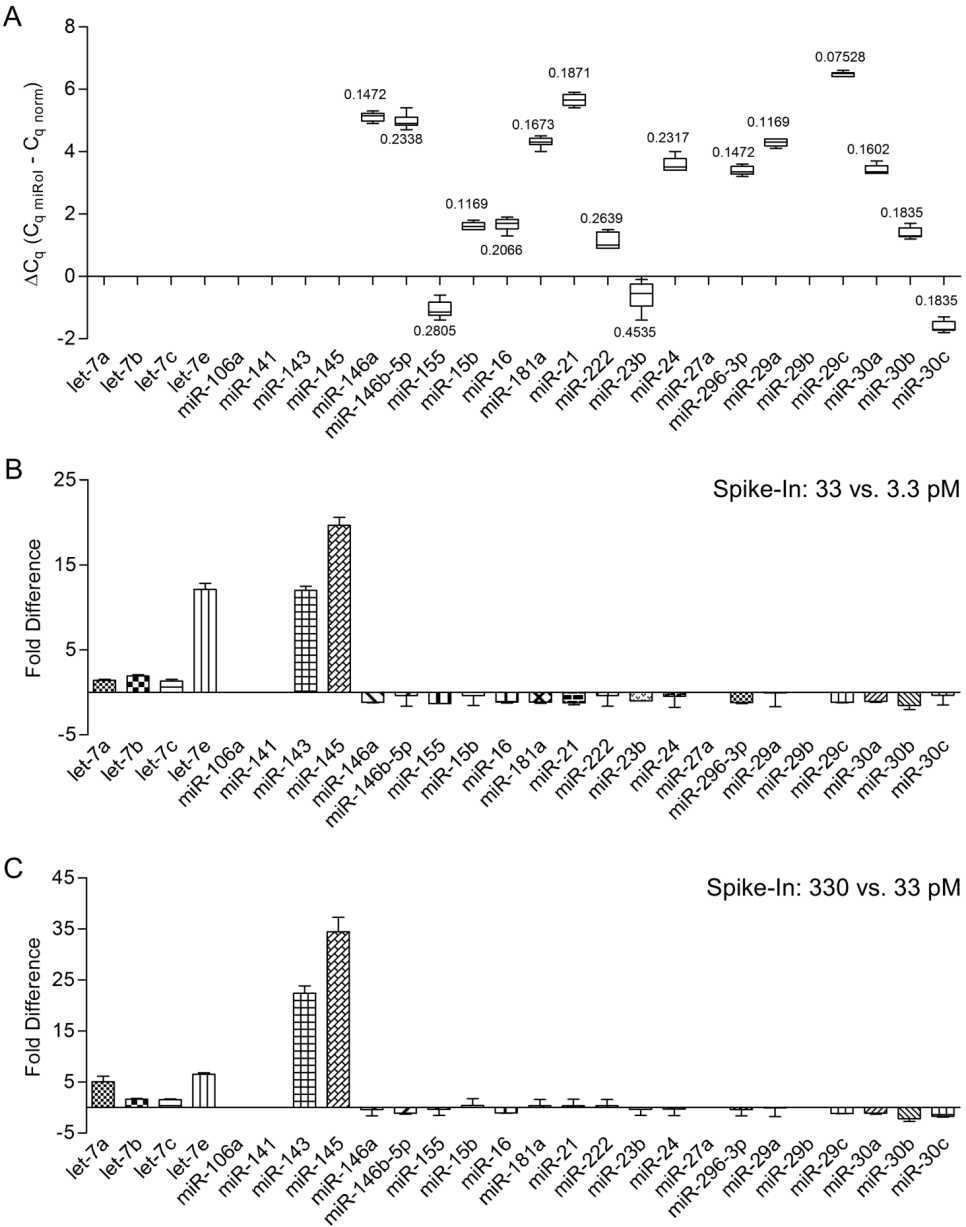
**Reproducibility and accuracy of miR-Q arrays**. Tukey box plots in Figure 4A show the inter-assay distribution of normalised values (ΔC_q _= C_q miRoI_-C_q norm_) of the 6 replicates. Each value was calculated as the mean of triplicate intra-assay measurements. Standard deviations (SD) of 6 replicates are given adjacent to the box plots. According to the MIQE guidelines we determined the accuracy of miR-Q arrays by spiking total RNA samples with 3.3, 33 and 330 pM synthetic miRNA (let-7e, miR-143 and miR-145). Fold differences between 3.3 and 33 pM (figure 4B) as well as 33 and 330 pM (figure 4C) were determined applying the ΔΔC_q _algorithm.

A measure for high reproducibility of RT-qPCR arrays is the evaluation of inter-assay variance. We assessed the reproducibility of miR-Q arrays by performing RT reaction from 6 independently differentiated THP-1 passages that were measured on 6 independent miR-Q array runs. For this purpose, THP-1 monocytes were differentiated into macrophages and after 28 h samples were taken and RT reactions were performed in parallel. This introduced experiment outlines a dual challenge to determine the reproducibility since independent biological samples were employed for determination of the inter-assay variance. Figure 4A shows the inter-assay distribution of normalised values (ΔC_q _= C_q miRoI _- C_q norm_) of 6 replicates each representing the mean of triplicate intra-assay measurement. As mentioned above the value C_q norm _was calculated as the geometric mean of the set of 5 stable reference snRNAs. In consistence with the data shown in Figure 2B, 10 miRNAs were not expressed in differentiated THP-1, or were below the detection limit. As shown by the box plots in Figure 4, the other 16 showed highly constant expression among the 6 biological replicates and independent runs, respectively. This particularly points out the high reproducibility of miR-Q arrays but also the studied biological system. For comparative analyses we highly recommend performance of simultaneous RT reactions of all samples included in a study using a thermal cycler. The preparation of master mixes also minimises inter-assay variance.

Additionally, we have performed spike-in experiments to check for the accuracy of the miR-Q assays. For this purpose, we have selected three miRNAs (let-7e, miR-143 and miR-145) that were proven to be absent in differentiated THP-1 (Figure 4A). RT reactions as performed for the validation of reproducibility were spiked with synthetic let-7e, miR-143 and miR-145 to give a final concentration in RT-qPCRs at 3.3, 33 and 330 pM, respectively. According to the MIQE guidelines, accuracy of a RT-qPCR refers to the difference between experimentally measured and actual concentrations as fold difference [16]. Accordingly, we performed miR-Q arrays for all concentrations and determined the fold differences between 3.3 and 33 pM as well as 33 and 330 pM applying the ΔΔC_q _algorithm, respectively. The reactions revealed 12 fold difference of both let-7e and miR-143 and 20 fold difference of miR-145 between 3.3 and 33 pM spike-in (Figure 4B). While detection of all other miRNAs remained unaffected, in the 33 pM spiked sample there was a slight cross reaction between let-7e spike-in and other let-7 family members resulting in 1.3 to 1.9 fold difference compared to the 3.3 pM control. Higher dosage of spike-in however led to a higher cross reaction within the let-7 family. As shown in Figure 4C, let-7e spike-in at a final concentration of 330 pM created 5 fold increased detection of let-7a and 6.5 fold detection of the specific target let-7e compared to the 33 pM spiked sample. Furthermore, there was a 22.4 fold difference of miR-143 and a 34 fold difference of miR-145 between both samples (Figure 4C). As we discussed earlier, the higher the concentration of the spike-in, the higher is the cross reaction between the assays and paralogous targets of a miRNA family. These data verify the accuracy of miR-Q arrays especially at low spike-in concentrations, which rather represent cellular and physiological miRNA concentrations compared to higher concentrations producing increased cross reactivity.

**Figure 5:**
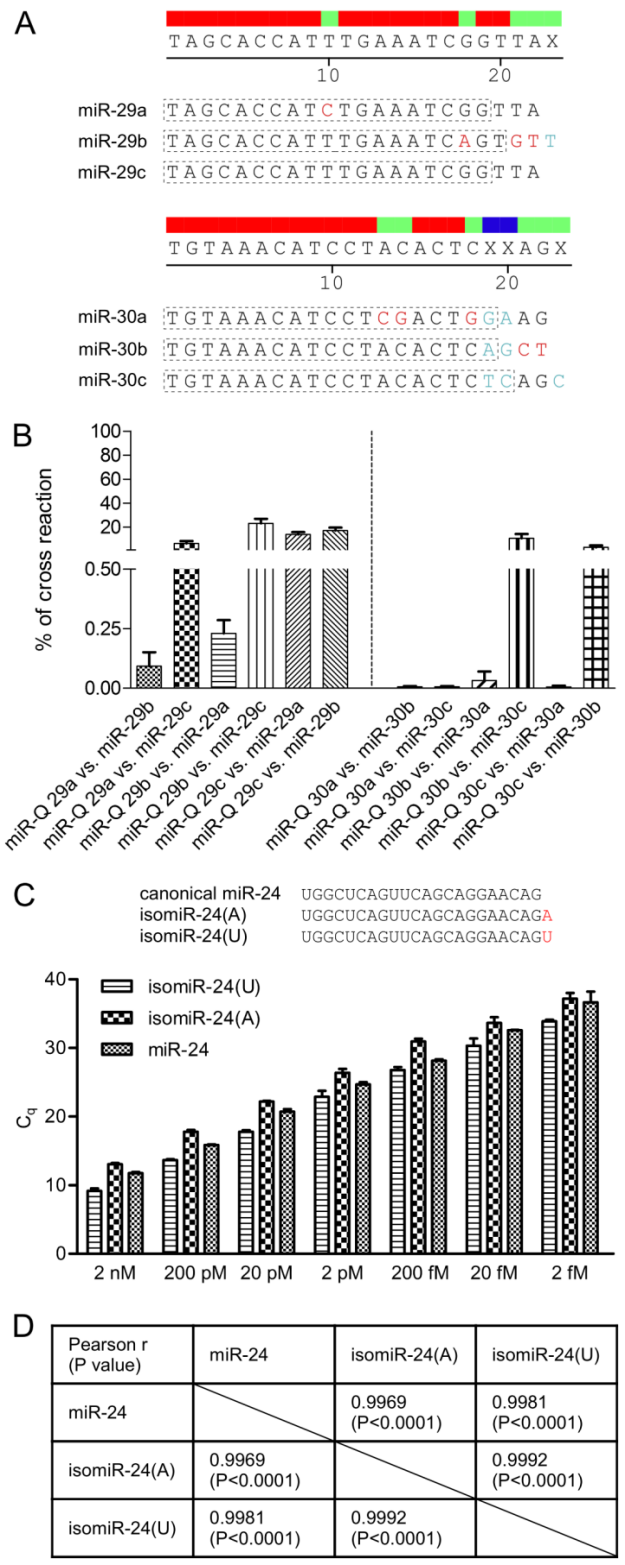
**Analytical specificity of miR-Q arrays**. Figure 5A shows the alignment of miR-29 as well as miR-30 family members. Dashed borders indicate binding sites of 'miRNA'-rev primers. The percentage of cross reaction is given between assay-specific and unspecific miRNA targets. Assay-specific targets represent the full value (Figure 5B). The design of the miR-Q approach allows simultaneous quantification of a canonical miRNA as well as its isomiRs as exemplified by miR-24 (Figure 5C). The columns in Figure 5C show triplicate determination of the linear range using synthetic miR-24 as well as synthetic isomiRs. Pearson correlation analysis revealed high linearity between all data sets possessing r < 0.9969 and two-tailed P values < 0.0001 (Figure 5D).

We have shown earlier that the miR-Q approach is able to accurately discriminate between the let-7 family members [9] pointing to the analytical specificity of this approach. Since many miRNA families exist and their members often possess few nucleotide exchanges, we examined the discriminative power of miR-Q by studying the intra-family-interaction exemplified by miR-29 and miR-30 family members. The cross reaction between the specific miR-Q and a paralogous miRNA target was determined along the entire dynamic range of assays. It was intriguing that a two nucleotide mismatch within the miRNA specific part of a 'miRNA'-rev oligonucleotide and a paralogous miRNA target resulted in less than 0.25% cross reactivity of assays to false targets. When the non-specific target differed only in one nucleotide the cross reaction varied between 10 and 20% (Figure 5A and 5B). This observation was true for the miR-29 as well as miR-30 family. Although the determined values point out the high target-specificity of miR-Q assays, in one mismatch cases it has to be considered that around 10% of the measured expression value may be derived from paralogous miRNAs of a family. Since the 'miRNA'-rev oligonucleotides (table 1) confer the specificity of miR-Q assays; the respective design determines the magnitude of cross reactivity and the analytical specificity. For minimising cross reactivity of miR-Q assays and if possible, we suggest designing mismatched base pairing at the 3' OH of the 'miRNA'-rev oligonucleotides. However, the respective target recognising part of the reverse primer has to be as long as possible to ensure proper hybridisation at 60°C. Nevertheless, while designing a new miR-Q assay, one has to take into account that an overlap of more than 3 nucleotides between RT6-'miRNA' and 'miRNA'-rev should be avoided because it results in high background of assays.

Recent deep sequencing efforts of small RNAs have shown that many mature miRNAs are either trimmed alternatively resulting in 5' and 3' variations or additional 3' non-template adenosines or uridines are transferred by nucleotidyl transferases post-transcriptionally [21]. These variants of the canonical miRNA are often referred to as 'isomiRs'. We were interested how far the miR-Q approach is biased toward detecting only the canonical mature miRNA without any additions or if it is possible to quantify the whole population of a given miRNA including isomiRs. Therefore, we performed additional experiments evaluating the bias of our approach by non-template adenosines or uridines present at the 3' end of non-canonical isomiRs based on miR-24 variants. Recently, this miRNA was shown to possess additional 3' adenosine or uridine modifications [22]. We performed comparative and paralleled spike-in experiments using synthetic canonical miR-24 as well as the A or U isomiRs, respectively. As it is shown in Figure 5C, the design of the miR-Q approach allowed the detection of isomiRs regardless of an additional adenosine or uridine. All variants were detected over the same linear range proving the coverage of the entire population. We outlined a correlation matrix by computing the Pearson correlation coefficient (r) for every pair of data sets. The analysis revealed high linearity between all data sets possessing r < 0.9969 and two-tailed P values < 0.0001 (Figure 5D). The miR-Q approach provides an advantage over other detection chemistries based e.g. on hairpin reverse primers allowing to capture the entire expression of a given miRNA including the canonical molecule as well as isomiRs.

### *Salmonella *infection of human macrophages results in specific miRNA response proven by miR-Q arrays

**Figure 6:**
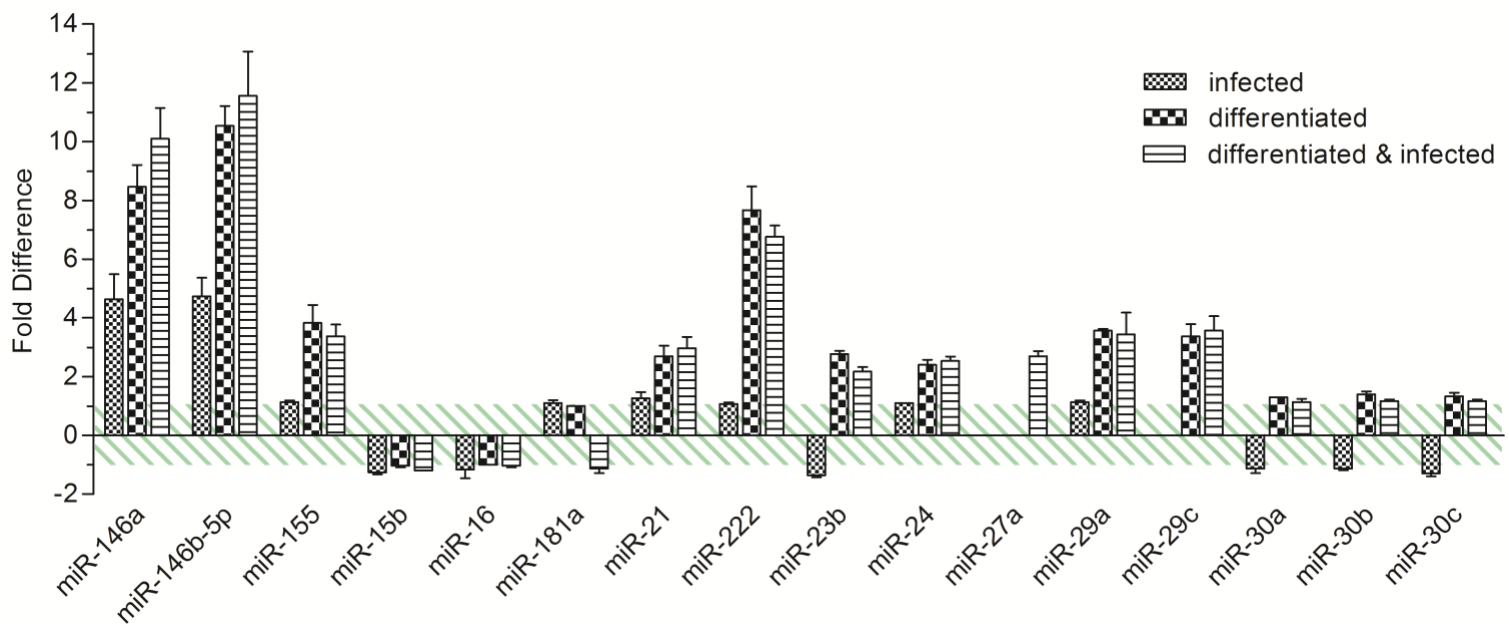
***Salmonella *infection of human monocytes reveals a specific miRNA response**. MiRNA regulation is shown in three groups using the monocytic THP-1 as a differentiation model. Fold differences between differently treated THP-1 and untreated controls are shown. Columns filled with small squares show monocytic THP-1 stimulated with viable *Salmonella*. Columns filled with big squares represent macrophages derived from PMA treated THP-1 and striped columns represent the third group being composed of differentiated macrophages that were infected with viable *Salmonella*. The bars indicate the SD of triplicate experiments each measured in triplicate. The green dashed area indicates balanced expression between samples and control.

The developed protocol was employed to investigate miRNA regulation in monocyte differentiation after PMA treatment as well as *Salmonella *infection. We studied miRNA regulation in three groups using the monocytic THP-1 as a differentiation model. The first group consisted of monocytic THP-1 cells that were stimulated with viable *Salmonella*. The second group represented macrophages derived from PMA treated THP-1, while the third was composed of differentiated macrophages that were infected with viable *Salmonella*. All groups were compared to the untreated controls performing triplicate measurements of triplicate biological samples. The correlation coefficients between the entire set of reference snRNAs of samples and controls ranged from 0.9884 to 0.9997 pointing out the high linearity and stability of the chosen reference snRNAs. All three treatments resulted in up-regulation of miR-146a/b from 5 to more than 10 fold (Figure 6), while only PMA treatment increased the miR-155 expression 3 fold. These results are consistent with the study of Taganov and colleagues, who reported comparable up-regulation of these miRNAs [23]. It furthermore underlines the accuracy of the presented protocol. Interestingly, miR-222, miR-21 and miR-24 were up-regulated after differentiation while 2 h stimulation of monocytes with viable *Salmonella *did not affect their expression (Figure 6). All three miRNAs were recently reported to play roles in monocyte differentiation into macrophages as well as dendritic cells [24,25]. While miR-23b possessed averaged 1.4 fold decreased expression 2 h post *Salmonella *infection of monocytic THP-1, PMA treatment increased its expression more than 2 fold in both groups. This is the first study reporting miR-23b dysregulation in monocyte stimulation and differentiation. Down-regulation of miR-23b was related to activation of the TGF-β1/Smad3 signalling pathway in hepatocytes [26]. TGF-β on the other hand is known to be a monocyte activator triggering cytokine production and mediating host defence [27]. Therefore, we hypothesise a miR-23b dependent TGF-β regulation in monocytes encountering pathogens or pathogen-associated molecular patterns (PAMPs) leading to host defence mechanisms. We found miR-27a to be only up-regulated in differentiated and infected THP-1. While miR-27a/b were recently reported to have antiviral effects in murine primary macrophages [28], this is the first study reporting increased expression of monocytic miR-27a upon *Salmonella *interaction. Accordingly, differentiation of THP-1 into macrophages led to more than 3 fold increased expression of miR-29a and miR-29c. The miR-29 family turns out to be a key regulator in host immune response to bacterial pathogens as it was recently shown to control the immune response to intracellular bacteria by targeting interferon-γ and to regulate apoptosis of primary human macrophages in mycobacterial infections [6,8].

## Conclusions

The presented approach is the first custom and non-commercial protocol for RT-qPCR arrays based on a miRNA specific RT-qPCR called miR-Q. The developed miR-Q arrays enable reliable and feasible quantification of small non-coding RNAs as exemplified by miRNA expression analysis during monocyte differentiation and infection. On one hand, well known miRNA dysregulation verified the accuracy of the presented method. On the other hand, several dysregulated miRNAs were newly identified in differentiated as well as infected human monocytes by means of the presented method and will provide a basis for our future studies.

## Methods

### Cell culture and RNA isolation

The human acute monocytic leukemia cell line THP-1 (DSMZ ACC 16) was cultured in suspension using RPMI 1640 (Biochrom AG) supplemented with 10% fetal bovine serum superior (Biochrom AG) and 10 μg/ml gentamicin (Biochrom AG) and was passaged twice weekly. THP-1 were in vitro differentiated into macrophages by adding 250 ng/ml phorbol-12-myristate-13-acetate (PMA). Differentiated cells were lysed after 28 h and total RNA was isolated using the miRVana Isolation Kit (Life Technologies). RNA integrity numbers (RIN) were determined using an Agilent 2100 Bioanalyzer and RNA Nano Chips (Agilent). All RNA isolations used in this study possessed RINs higher than 9.

### Infection experiments

Monocytic THP-1 were seeded in 6-well plates without gentamicin at a density of 5 × 10^5 ^cells per well. Afterwards, they were stimulated with viable *Salmonella *serovar Typhymurium DT104 at a multiplicity of infection (MOI) of 10 in triplicate. 2 h post stimulation adherent as well as non-adherent cells were washed twice with warm PBS and lysed for total RNA isolation. Non-treated THP-1 served as controls. Macrophages derived from monocytic THP-1 were also infected with *Salmonella *serovar Typhymurium DT104. For this purpose, 3.5 × 10^5 ^cells per well were seeded in 6-well plates and differentiated as described above. Macrophages remained either non-infected or were infected with exponential-phase *Salmonella *serovar Typhymurium DT104 at an MOI of 10. 2 h post infection supernatants were removed and adherent cells were washed twice with warm PBS. Adherent macrophages were lysed and total RNA was isolated as described above.

### Oligonucleotides

All miRNA specific oligonucleotides for RT as well as qPCR were designed as described earlier [9] and were synthesised and HPLC purified by Metabion AG and Eurogentec Deutschland GmbH. The miRNA sequences were taken from the miRNA database miRBase 17 [29].

### Individual and multiplexed reverse transcription

RT of miRNAs for single RT-qPCR was performed as described earlier [9]. For multiplexed RT, a pool of miRNA- as well as reference snRNA-specific DNA-oligonucleotide (RT6-'miRNA' or RT6-'snRNA' and the RevertAid™ M-MuLV Reverse Transcriptase (Fermentas GmbH) were employed to transcribe miRNAs and reference snRNAs into cDNA. The RT-6-Pool represented a blend of DNA-oligonucleotides specific for 26 miRNAs as well as 5 snRNAs serving as reference genes for normalisation. The final concentration of the RT-6-Pool was 1 μM representing 32.3 nM of each DNA-oligonucleotide. The reaction was performed in 200 μl PCR tubes using the Veriti^® ^Thermal Cycler (Life Technologies). Between 500 ng and 1 μg total RNA and 1 μl of the RT-6-Pool was first prepared in a 5 μl volume. The mixture was incubated at 70°C for 5 min and chilled on ice. After adding 5× RT-Buffer, 1 mM dNTPs and 200 U Reverse Transcriptase, the volume was adjusted to 10 μl by adding nuclease-free water. The reaction was started at 37°C for 5 min followed by 42°C for 2 h. The enzyme was inactivated by heating at 95°C for 5 min. Afterwards the cDNA pool was cooled down for at least 1 min on ice and 90 μl of nuclease-free water was added yielding the template for qPCR arrays. RT reactions serving for comparative analyses were always run in parallel using identical amounts of total RNA.

### RT-qPCR arrays and conventional miR-Q

Arrayed triplicate measurement of 26 miRNAs and 5 reference snRNAs was performed in 10 μl final reaction volume by means of SYBR Green detection chemistry using the SensiMix SYBR Hi-ROX Kit (Bioline GmbH) and the Step One Plus Cycler (Life Technologies). All reactions were carried out using clear MicroAmp^® ^Fast 96-Well Reaction Plates (Life Technologies) that were sealed with adhesive films. Firstly, a master mix was prepared in a 945 μl volume consisting of 2× SensiMix SYBR Hi-ROX, 100 nM of each universal primer (MP-fw and MP-rev) and 100 μl of diluted RT-reaction. After dispensing 9 μl of the master mix in wells of a 96 well plate, 1 μl of each miRNA- or snRNA-specific reverse primer ('miRNA or snRNA'-rev) was added to respective wells of the array at a final concentration of 5 nM. The amplification was carried out via the first step at 95°C for 10 min, followed by 40 cycles with 15 s at 95°C, 20 s at 60°C and 20 s at 72°C. The fluorescence signal was acquired at 60°C. Quality control was performed by subsequent melting curve analysis by 95°C for 1 min, 60°C for 2 min, ramping from 60°C to 95°C at 2°C/min and acquiring the signal. Samples containing water instead of the specific 'miRNA or snRNA'-rev primer served as negative controls (NC). Conventional miR-Q was performed as described previously [9].

### Normalisation of miRNA expression and comparative quantification

Normalisation of miRNA expression was performed using a set of snRNAs: SNORD44 (NR_002750.2), SNORD47 (NR_002746.1), SNORD48 (NR_002745.1), SNORD52 (NR_002742.2) and RNU6 (NR_004394). After calculating the C_q _mean of each reference snRNA, the C_q _geometric mean of all reference snRNAs was used to normalise the miRNA expression values. The difference between the C_q _of the miRNA of interest (C_q miRoI_) and the calculated geometric mean (C_q norm_) was calculated yielding the ΔC_q sample _or ΔC_q calibrator_, respectively. The relative quantification (ΔΔC_q_) was performed by determining the difference between ΔC_q sample _and ΔC_q calibrator_. Fold differences were determined by calculating 2 to the power of-ΔΔC_q_.

## Competing interests

The authors declare that they have no competing interests.

## Authors' contributions

SS conceived the study, performed experiments and analyses, wrote and edited the manuscript. JS contributed to the experiment design, data analysis and writing of the manuscript. LH and MB performed experiments. RE contributed to the writing of the manuscript. All authors read and approved the final manuscript.

## Supplementary Material

Additional file 1 **Quality control of presented miR-Q assays**. Calculated parameters (e.g. slopes, Y-intercepts and r^2^) for all presented miR-Q assays are given according to the MIQE guidelines. Click here for file
